# Zusammenhänge zwischen genetischen Ursachen psychischer Erkrankungen und seltenen monogenetischen Epilepsien

**DOI:** 10.1007/s00115-026-01982-3

**Published:** 2026-05-29

**Authors:** Caroline Baumgartner, Tobias Baumgartner, Alexandra Philipsen, Rainer Surges, Eva C. Schulte

**Affiliations:** 1 Klinik für Psychiatrie und Psychotherapie, Universitätsklinikum Bonn, Venusberg-Campus 1, 53127 Bonn, Deutschland; 2 Universitätsklinikum Bonn, Klinik und Poliklinik für Epileptologie, Universität Bonn, Bonn, Deutschland; 3 Universitätsklinikum Bonn, Institut für Humangenetik, Universität Bonn, Bonn, Deutschland; 4 LMU Klinikum, Institut für Psychiatrische Phänomik und Genomik (IPPG), LMU München, München, Deutschland; 5 Deutsches Zentrum für Psychische Gesundheit (DZPG), Standort München-Augsburg, München-Augsburg, Deutschland

**Keywords:** Epilepsie, Genetik, Entwicklungsbedingte Enzephalopathien, Epileptische Enzephalopathien, Zielgerichtete Therapie, Epilepsy, Genetics, Developmental encephalopathy, Epileptic encephalopathy, Targeted therapy

## Abstract

Psychische Erkrankungen treten bei Menschen mit Epilepsie häufiger auf, umgekehrt haben psychiatrische Patient*innen ein erhöhtes Epilepsierisiko. Psychosoziale Faktoren, Medikamenteneffekte, peri- und interiktale Symptome spielen ebenso wie gemeinsame genetische Mechanismen eine Rolle, insbesondere bei seltenen monogenetischen Epilepsiesyndromen. Der Übersichtsartikel behandelt exemplarisch *GRIN2A*-assoziierte Syndrome, entwicklungsbedingte und epileptische Enzephalopathien einschließlich Dravet-Syndrom sowie tuberöse Sklerose. Zusammengefasst werden neuropsychiatrische Manifestationen, pathophysiologische Grundlagen und zielgerichtete Therapien. Schwerpunkt ist die systematische Erfassung psychiatrischer Symptome und die präzise klinische Phänotypisierung zur Beurteilung krankheitsmodifizierender Therapieeffekte jenseits der Anfallskontrolle. Ziel ist es, klinische Implikationen, Versorgungsdefizite und offene Fragen aufzuzeigen und interdisziplinäre neurologisch-psychiatrische Betreuung zu fördern.

**Zusatzmaterial online:**

Die Online-Version dieses Beitrags (10.1007/s00115-026-01982-3) enthält eine Tabelle zur beispielhaften Darstellung psychiatrischer und neurologischer Manifestationen seltener genetischer Erkrankungen.

## Abstract (en)

Psychiatric disorders are more common in people with epilepsy and vice versa psychiatric patients have an increased risk of epilepsy. Psychosocial factors, medication effects, peri-ictal or interictal symptoms play a role as do shared genetic mechanisms, particularly in rare monogenic epilepsy syndromes. This review article exemplary discusses *GRIN2A*-associated disorders, development-related and epileptic encephalopathies including Dravet syndrome and tuberous sclerosis complex. Neuropsychiatric manifestations, pathophysiological foundations and targeted treatment are summarized. Emphasis is placed on the systematic assessment of psychiatric symptoms and detailed clinical phenotyping to evaluate disease-modifying effects of emerging therapies beyond seizure control. The article highlights the clinical implications, gaps in care and to present open questions and to promote interdisciplinary neurological psychiatric management.

## Hintergrund

Menschen mit psychischen Erkrankungen weisen ein erhöhtes Risiko auf, im Verlauf des Lebens eine Epilepsie zu entwickeln [6]. Umgekehrt treten bei Menschen mit Epilepsie psychische Erkrankungen mit einer Lebenszeitprävalenz von 35 % auf. Depressionen und Angststörungen treten etwa doppelt so häufig, Autismusspektrumstörungen (ASS) oder Psychosen [27, 45] häufiger auf als in der Allgemeinbevölkerung. Krankheitsassoziierte psychosoziale Belastungen tragen zur Entwicklung psychischer Erkrankungen bei. Menschen mit Epilepsie können in Berufswahl, Teilnahme am Straßenverkehr und Ausübung bestimmter Freizeitaktivitäten eingeschränkt sein und von Stigmatisierung im sozialen Umfeld betroffen sein [31]. Anfallssuppressiva können psychiatrische Symptome auslösen oder verstärken [48]. Inter- und periktale psychiatrische Symptome, die zumeist nicht die Diagnosekriterien einer psychischen Erkrankung erfüllen, sind bei Epilepsien beschrieben [11].

Gemeinsame genetische Faktoren spielen eine Rolle bez. Des gehäuften Vorkommens psychischer Erkrankungen bei Menschen mit Epilepsie. In genomweiten Assoziationsstudien konnten gemeinsame prädisponierende genetische Veränderungen identifiziert werden [22]. Im Bereich seltener genetischer Risikofaktoren mit hohen Effektstärken gibt es eine Reihe an Genen, in denen genetische Varianten für psychische Erkrankungen und Epilepsien prädisponierend sind [15, 44], so führen z. B. Varianten des *PCDH19*-Gens zu einer epileptischen Enzephalopathie, die mit erhöhtem Risiko (13 %) für spätere psychische Erkrankungen, insbesondere psychotische Störungen assoziiert ist [39].

Epilepsien sind heterogene, meist multifaktoriell bedingte Erkrankungen, doch sind genetische Faktoren an der Entstehung des Großteils der Epilepsien, inkl. der erworbenen Epilepsien [15], beteiligt. Das Spektrum genetischer Faktoren reicht von häufigen genetischen Varianten mit geringen Effekten auf den Phänotyp (sog. Einzelnukleotidpolymorphismen) bis zu monogenetischen Erkrankungen, bei denen eine einzelne genetische Veränderung mit sehr starkem Effekt ausreichend ist, um die Erkrankung hervorzurufen [40]. Insbesondere bei entwicklungsbedingten- und epileptischen Enzephalopathien („developmental and epileptic encephalopathies“, DEE) wurden in den letzten Jahren durch den Einsatz moderner Sequenziertechnologien bei bis zu 50 % monogenetische Ursachen identifiziert [37]. Hier treten neuropsychiatrische Störungen wie ASS [45] besonders häufig auf. Mit wachsendem Verständnis pathophysiologischer Mechanismen stehen für eine zunehmende Zahl seltener, monogenetischer Epilepsien krankheitsspezifische, teils pathophysiologisch geleitete Therapieansätze zur Verfügung [3], die auf die Reduktion der Anfallsfrequenz abzielen, zunehmend aber auch epilepsieassoziierte und psychiatrische Komorbiditäten adressieren. Obwohl psychiatrische Störungen bei vielen seltenen genetischen Epilepsien bekannt sind, werden diese bislang selten systematisch erfasst. Eine verbesserte Phänotypisierung ist essenziell, um den Einfluss zielgerichteter Therapien auf psychische Symptome valide beurteilen zu können.

Im Folgenden werden exemplarisch seltene monogenetische Erkrankungen mit Epilepsie und psychiatrischer Symptomatik dargestellt (s. auch Tabelle Zusatzmaterial online). Ziel ist ausdrücklich keine vollständige Darstellung des gesamten Themenfeldes, sondern die Hervorhebung praxisrelevanter Aspekte, offener Fragestellungen und Versorgungsbedarfe.

## *GRIN2A-*assoziierte Syndrome

Den meisten psychischen Störungen liegt ein polygenes Vererbungsmuster zu Grunde, doch konnte gezeigt werden, dass der heterozygote Funktionsverlust des *GRIN2A*-Gens mit einem fast 20fach erhöhten Schizophrenierisiko einhergeht [49] und damit eine seltene, aber nahezu „monogenetische“ Ursache einer Schizophrenie darstellen könnte. Zur Penetranz existieren noch keine systematischen Analysen.

Varianten in *GRIN2A* können zu (psycho-)motorischen Entwicklungsstörungen, Intelligenzminderung (ID) und verschiedenen Epilepsiesyndromen führen und mit psychischen Erkrankungen wie Depression und Angststörung einhergehen. Während mit Schizophrenie nur *GRIN2A*-Null-Varianten signifikant assoziiert sind, finden sich bei Patient*innen mit Epilepsie und Entwicklungsstörungen *GRIN2A*-Null- und *GRIN2A*-Missense-Varianten [29, 43]. In der Kohorte von Lemke et al. wurden 84 Patient*innen mit *GRIN2A*-Null-Variante identifiziert, dabei 23 mit psychischen Erkrankungen (u. a. affektive und Angststörungen, Psychosen, Persönlichkeitsstörungen). Interessanterweise manifestierte sich die Schizophrenie bei *GRIN2A*-Null-Varianten bereits im frühen Kindes- oder Jugendalter. *GRIN2A* kodiert für die GluN2A-Untereinheit des N‑Methyl-D-Aspartat(NMDA)-Rezeptors. *GRIN2A*-Null-Varianten führen durch kompletten Funktionsverlust zu einer gestörten NMDA-Rezeptor-Funktion, was zur Hypothese der glutamatergen Unterfunktion bei Schizophrenie passt [9] und im Einklang mit der Rolle glutamaterger Systeme bei Epilepsien steht [8]. In einer kleinen Fallzahl (*N* = 4) konnte gezeigt werden, dass L‑Serin (koagonistischer Effekt auf den NMDA-Rezeptor) zur Verbesserung neuropsychiatrischer Symptome (Reduktion psychotischer Symptomatik/Verhaltensstörungen) führte [29]. *GRIN2A*-assoziierte Störungen verdeutlichen, dass Veränderungen eines einzelnen Rezeptorproteins je nach funktioneller Ausprägung zu einem breiten Spektrum neuropsychiatrischer Störungen führen können, womit sie gemeinsame genetische und pathophysiologische Mechanismen unterschiedlicher Krankheitsbilder widerspiegeln.

## Entwicklungsbedingte und epileptische Enzephalopathien

Entwicklungsbedingte und epileptische Enzephalopathien (DEE) stellen eine Gruppe schwer behandelbarer Epilepsiesyndrome dar, gekennzeichnet durch pharmakoresistente Anfälle sowie Entwicklungsstillstand/-rückgang, überwiegend im Säuglings- und Kleinkindalter auftretend [41]. Bis zum 16. Lebensjahr manifestiert sich eine DEE bei ca. einem von 590 Kindern [38]. Die epileptischen Anfälle und die interiktuale epileptiforme Aktivität und die zugrunde liegende Ätiologie tragen zur Entwicklungsstörung bei [41]. Besonders häufig finden sich ASS [45], wo für einzelne DEE-assoziierte Gene (z. B. *SCN1A*) Prävalenzen von > 50 % beschrieben sind. Weitere häufige Komorbiditäten sind affektive und Angststörungen sowie psychotische Symptome [41].

Die genetischen Ursachen der DEE sind heterogen; mithilfe moderner Sequenzierungstechnologien wurden > 900 ursächliche Gene identifiziert [35]. Für einzelne DEE liegen spezifische Therapien vor, wie ketogene Diät beim GLUT1-Defizit-Syndrom [33], die Anzahl zielgerichteter Therapien wird in den kommenden Jahren voraussichtlich zunehmen [47].

### Dravet-Syndrom

Exemplarisch ist das Dravet-Syndrom (DS) als eine der häufigsten DEE zu nennen. In 85 % der Fälle liegt eine *SCN1A*-Haploinsuffizienz zugrunde [2]. Die genetischen Varianten treten meist de novo auf mit Erstmanifestation zwischen dem 4. und 12. Lebensmonat mit fieberassoziierten epileptischen Anfällen. Typischerweise treten seitenwechselnde (hemi-)klonische oder generalisiert tonisch-klonische Anfälle auf, häufig fieberassoziierte Status epileptici [13]. Patient*innen haben ein erhöhtes SUDEP-Risiko („sudden unexpected death in epilepsy“; [18]). In der zweiten Lebensdekade nimmt die Anfallsfrequenz und Neigung zu fieberassoziierten Status epileptici i. d. R. ab. Im Erwachsenenalter treten Anfälle typischerweise als schlafgebundene tonisch-klonische Anfälle auf [16]. Entwicklungsverzögerung mit Verhaltensauffälligkeiten, ADHS sowie ASS treten nach dem 1. Lebensjahr bei zuvor regelrecht entwickelten Kindern auf; moderate bis schwere ID ist häufig [42]. Es entwickeln sich Gang- und motorische Störungen. Erwachsene mit DS zeigen überwiegend eine ID (86 %), in 60 % Symptome einer Aufmerksamkeitsdefizit-Hyperaktivitäts-Störung (ADHS). Bei ca. einem Drittel der Patient*innen werden Angststörungen oder psychotische Symptome beschrieben, bei ca. 30 % eine ASS, affektive Störungen bei 10–14 %. Im Verlauf stehen häufig Verhaltensauffälligkeiten und motorische Einschränkungen stärker im Vordergrund [20, 42].

Zur Behandlung werden breitwirksame anfallssuppressive Medikamente (Valproat, Levetiracetam, Clobazam, Bromid) eingesetzt. Stiripentol, Cannabidiol und Fenfluramin sind in Deutschland als Zusatztherapie zugelassen. Fenfluramin zeigte in Studien eine signifikante Reduktion der Anfallshäufigkeit [28] und könnte positive Effekte auf Kognition und Aufmerksamkeit haben [4, 21]. Die Wirksamkeit auf weitere psychische Symptome ist noch nicht untersucht. Klassische Natriumkanalblocker (z. B. Carbamazepin, Lamotrigin, Phenytoin) sollten vermieden werden. Sie können zur Verschlechterung der Anfallssituation führen. Ketogene Diät und Vagusnervstimulator sind weitere therapeutische Optionen [7].

Vor Kurzem wurde eine Screeningcheckliste entwickelt, die systematisch Komorbiditäten erfasst (Dravet Syndrome-Associated Neuropsychiatric Comorbidities, DANCE; [17]), einsetzbar um den Effekt therapeutischer Interventionen auch auf psychische Symptome besser evaluieren zu können.

## Tuberöse-Sklerose-Komplex-Erkrankung

Die Tuberöse-Sklerose-Komplex(TSC)-Erkrankung ist i. d. R. durch Varianten im *TSC1*-(ca. 25 %) oder *TSC2*-Gen (ca. 70 %) bedingt, die zu Überaktivierung des mTOR-Komplexes führen. Es kann zu Fehlbildungen und Tumoren des Gehirns (Hamartome, subependymale Riesenzellastrozytome [SEGA]), der Niere, des Herzens und anderer Organsysteme sowie Angiofibromen des Gesichtes kommen. 80–90 % der Betroffenen leiden an früh beginnender Epilepsie. TSC-assoziierte neuropsychiatrische Störungen (TSC-Associated Neuropsychiatric Disorders, TAND) bestehen in 90 % der Fälle [30]. ASS treten in 26–45 % auf [46]. Neuropsychiatrische Störungen sind häufig unterdiagnostiziert und -behandelt trotz erheblicher Krankheitslast. Erhöhtes Risiko für TAND besteht bei Varianten in *TSC2. *Weitere Risikofaktoren sind epileptische Anfälle und ID. Typische Bereiche mit psychosozialen Schwierigkeiten werden zu „Clustern“ zusammengefasst (Stimmung/Ängste, Essen/Schlafen, Verhaltensdysregulation, ASS, neuropsychologische Einschränkungen, Schwierigkeiten in akademischer Ausbildung, Hyperaktivität/Impulsivität; [46]). Stehen im Kindes- und Jugendalter bei Patient*innen mit schwerer ID Sprachverzögerung sowie repetitives/ritualistisches Verhalten, Hyperaktivität/Impulsivität und Schlafstörungen im Vordergrund, treten im Erwachsenenalter vermehrt Depressionen und Angststörungen auf [12].

Mit dem mTor-Inhibitor Everolimus liegt für TSC eine spezifische Therapie vor zur Behandlung der SEGA und renaler Angiomyolipome [34], sie kann zudem zur Behandlung pharmakoresistenter Epilepsie [36] bei TSC eingesetzt werden. Es gab Hinweise auf positive Effekte von mTor-Inhibitoren auf neuropsychiatrische Symptome im Mausmodell [24]. Zur Wirkung auf psychiatrische Symptome beim Menschen ist die Datenlage heterogen: Im Kindes‑/Jugendalter fanden sich in zwei Studien keine positiven Effekte auf TAND [24, 36]. In einer weiteren Studie wurden bei Anfallsreduktion auch positive Effekte auf die „quality of life“ und Verhaltensauffälligkeiten [26] beschrieben. Bei Erwachsenen zeigte sich eine Besserung von ASS in der japanischen EXIST-3-Studie [32]. Ob eine zielgerichtete Therapie in der präsymptomatischen Phase neuropsychiatrischen Symptomen vorbeugen kann, wird aktuell im Rahmen der PROTECT-Studie untersucht [14].

## Schlussfolgerung

Monogenetische Epilepsien, die häufig zu schweren Entwicklungsstörungen führen, sind oft mit einer Vielzahl psychischer Erkrankungen wie schizophreniformen Störungen, ASS, ADHS-ähnlicher Symptomatik und Depressionen assoziiert. Auch wenn es sich per definitionem um seltene Erkrankungen/Epilepsien handelt, ist aufgrund der Vielzahl identifizierter Gene die Wahrscheinlichkeit, eine genetische Ursache einer Epilepsie zu identifizieren, in bestimmten klinischen Konstellationen – wie begleitende Entwicklungsstörung, ASS oder positive Familienanamnese – gar nicht so selten. Diese Patientengruppen benötigen eine multiprofessionelle Versorgung. Psychiatrische Symptome haben in allen Altersbereichen eine wichtige Bedeutung für die Lebensqualität der Betroffenen und müssen stärker beachtet werden. Die frühzeitige, korrekte Erkennung psychiatrischer Symptome ist wichtig, um eine optimale Behandlung zu gewährleisten. Die Kontinuität psychiatrischer Betreuung im Rahmen der Transition von der Kinder- und Jugendmedizin in den Erwachsenenbereich ist eine Herausforderung. Unzureichende Strukturen zur interdisziplinären Behandlung und teils Vorbehalte der Behandelnden aufgrund der komplexen Erkrankungsbilder erschweren eine kontinuierliche Versorgung. Die wenigen hierzu existierenden Daten für einzelne Syndrome legen jedoch nahe, dass bis auf ggf. geringere Zieldosen und langsameres Eindosieren keine großen Unterschiede in der Behandlung von Patient*innen mit seltenen genetischen Epilepsien und psychischen Erkrankungen gegenüber denen ohne eine seltene genetische Erkrankung bestehen (vgl. [5]; sowie Radtke et al. [in dieser Ausgabe von *Der Nervenarzt*]). Die Indikation zur medikamentösen Behandlung einer psychischen Erkrankung bei Menschen mit Epilepsie sollte ebenso gestellt werden wie bei Betroffenen ohne Epilepsie. Die Therapie sollte nicht aus Angst vor Verschlechterung der Anfallssituation vorenthalten werden [19] – der Einsatz der meisten modernen Neuroleptika und Antidepressiva ist im moderaten Dosisbereich relativ unproblematisch [10]. Ausnahmen stellen u. a. Clozapin oder Bupropion dar [2]. Pharmakologische Interaktionen zwischen Anfallssuppressiva und Psychopharmaka müssen berücksichtigt werden.

Angesichts der zunehmenden Entwicklung spezifischer pharmakologischer und gentherapeutischer Ansätze kommen der frühzeitigen molekulargenetischen Diagnostik und standardisierten Erfassung psychiatrischer Symptome eine zentrale Bedeutung zu, um krankheitsmodifizierende Effekte über die Anfallskontrolle hinaus beurteilen zu können. Für die psychiatrische Praxis bedeutet dies, bei Patient*innen mit o. g. psychischen Symptomen und epileptischen Anfällen – aktuell oder in der Vergangenheit – hellhörig zu werden, nach molekulargenetischer Diagnostik zu fragen, Patient*innen zur genetischen Beratung und ggf. Testung an eine Spezialambulanz zu vermitteln oder selbst eine genetische Diagnostik einzuleiten (s. hierzu auch [23]).

## Fazit für die Praxis

- Monogenetische Epilepsien sind häufig mit einer Vielzahl psychischer Erkrankungen assoziiert. Bei Patient*innen mit epileptischen Anfällen und psychischen Erkrankungen wie schizophreniformen Störungen, Autismusspektrumstörungen, ADHS(Aufmerksamkeitsdefizit-Hyperaktivitäts-Störung)-ähnlicher Symptomatik sowie Depressionen gilt es daher, hellhörig zu werden und eine molekulargenetische Diagnostik zu erfragen bzw. diese einzuleiten.
- Die wenigen zur Behandlung existierenden Daten legen nahe, dass bis auf ggf. geringere Zieldosen und langsameres Eindosieren keine großen Unterschiede in der Behandlung von Patient*innen mit seltenen genetischen Epilepsien und psychischen Erkrankungen gegenüber denen ohne eine seltene genetische Erkrankung bestehen.
- Die Indikation zur medikamentösen Behandlung einer psychischen Erkrankung bei Menschen mit Epilepsie sollte ähnlich gestellt werden wie bei Betroffenen ohne Epilepsie. Der Einsatz der meisten modernen Neuroleptika und Antidepressiva ist im moderaten Dosisbereich relativ unproblematisch. Ausnahmen stellen u. a. Clozapin oder Bupropion dar.
- Eine interdisziplinäre Behandlung der Patient*innen mit seltenen monogenetischen Epilepsien und psychischen Erkrankungen ist anzustreben.

## Supplementary Information

Tabelle e1: Beispielhafte Darstellung psychiatrischer und neurologischer Manifestationen seltener genetischer Erkrankungen

## Data Availability

Alle dieser Arbeit zugrunde liegenden Daten sind in diesem Artikel enthalten.
