# Supplementary material for: Associations Between Genetic Causes of Psychiatric Disorders and Rare Monogenic Epilepsies
Source: Nervenarzt. 2026 May 29;97(4):345–50. [Article in German] doi: 10.1007/s00115-026-01982-3 (PMC13315162; doi:10.1007/s00115-026-01982-3)
Supplement: Supplementary file 1 — Tabelle e1: Beispielhafte Darstellung psychiatrischer und neurologischer Manifestationen seltener genetischer Erkrankungen [file 115_2026_1982_MOESM1_ESM.pdf]

Beispielhafte Darstellung psychiatrischer und neurologischer Manifestationen seltener genetischer Erkrankungen

Syndrome/Erkrankungen	Genetische Veränderungen	Neurologische/epileptologische Manifestationen	Neuropsychiatrische Manifestationen	Therapie
***GRIN2A*-assoziierte Syndrome**<br>*(Farsi 2024; Shepard 2024; Strehlow 2024, Lemke et al., 2025, Singh et al. 2022)*	*Pathogene Einzelnukleotidvarianten (engl. single nucleotide variant; SNVs) in GRIN2A*	Beginn im 3-6 Lebensjahr; assoziiert mit einem Spektrum fokaler Epilepsiesyndrome: selbst-limitierende Epilepsie mit zentrotemporalen Spikes (Rolando-Epilepsie) , DEE mit kontinuierlicher Spike Wave Entladungen im Schlaf (incl. Landau-Kleffner-Syndrom)	Schizophrenie selten auch ohne ID oder Epilepsie auftretend; affektive Störungen, Angststörungen, Sprachentwicklungsstörungen, Bewegungsstörungen, ID, aber auch Pat. mit normaler Intelligenz	Symptomorientierte Therapie (ASM) /Behandlungsversuche mit L-Serin (bei null-Varianten) als NMDAR-Co-Agonist
**Dravet -Syndrom**<br>*(Dravet 2011; Huang et al. 2021; Selvarajah et al. 2025; Lagae et al. 2019; Giorgi et al. 2024)*	*Pathogene SNVs in SCN1A* (85%), *vereinzelt auch PCDH19, STXBP1, SCN2A*	Beginn im 1. Lebensjahr; fieberassoziierte Anfälle; hemiklonische und generalisierte tonisch-klonische Anfälle; Risiko für Status epilepticus; erhöhtes SUDEP-Risiko; Entwicklung einer progredienten Gangstörung	ADHS (~60 %), ASS (~30 %), psychotische Symptome (≈ 1/3 der Erwachsenen), Impulsdurchbrüche, Depressionen, Angststörungen, Schlafstörungen	Valproat, Clobazam, Bromid; Stiripentol, Cannabidiol, Fenfluramin, ketogene Diät, Vagusnervstimulation/ Vermeidung von Natriumkanalblockern
***CDKL5*-assoziierte Syndrome**<br>(*Benke 2024; Leonard 2022)*	*Pathogene SNVs in CDKL5*	Anfälle beginnen in der Regel in den ersten 6 Monaten, epileptische Spasmen, tonische, myoklonische und tonisch-klonische Anfälle, Großteil der Patient*innen nicht gehfähig, keine verbale Kommunikation	Schwere Entwicklungsstörung/ID, Schlafstörungen, Autismus (Stereotypien)	Symptomorientierte Therapie, ASM, ketogene Diät
**Tuberöse Sklerose Komplex *(TSC)***<br>*(de Vries et al. 2018/2023; Northrup et al. 2021; Krueger et al. 2013/2017; Mizuguchi et al. 2019; Driedger et al. 2025)*	*Pathogene SNVs in TSC1* (25%), *TSC2* (70%)	Häufig früher Beginn therapierefräkterer Anfälle/infantiler Spasmen	TSC-assoziierte neuropsychiatrische Störungen (TAND): Autismus-Spektrum-Störungen; ADHS; Angststörungen; Depressionen; Verhaltensdysregulation; Schlaf- und Essstörungen (Lebenszeitprävalenz ~90 %)	ASM (u.a. Vigabatrin)/ Everolimus (mTOR-Inhibitor) bei SEGA, Angiomyolipomen und Epilepsie; heterogene Ergebnisse bzgl. Wirksamkeit im Bezug auf psychiatrische Symptome, epilepsiechirurgische Therapie
**GATOR1-assoziierte Syndrome**<br>*(Baulac, Baldassari 2023; Moloney 2023*	*Pathogene SNVs in DEPDC5, NPRL2, NPRL3*	Verschiedene familiäre fokale Epilepsiesyndrome, teils auch DEE.<br>FCD bei einem Teil der Patienten im zerebralen MRT nachweisbar.	Intelligenz i.d.R. nicht beeinträchtig, teils jedoch auch Entwicklungsverzögerung, ASS und andere psychiatrische Symptome	Epilepsiechirurgische Therapie bei Nachweis einer FCD/ experimenteller Einsatz von mTOR-Inhibitoren
***MEF2C*-assoziiertes Syndrom**<br>*(Coleman et al. 2024)*	*Pathogene SNVs in MEF2C*	Fieberkrampfanfälle, infantile Spasmen, generalisierte tonisch-klonische, myoklonische und fokale Anfälle	Neurobehaviorale/psychiatrische Symptome, einschließlich autistischer Merkmale (verminderte soziale Interaktion, stereotype Bewegungen, insbesondere der Hände, wiederholtes Schaukeln und Kopfschütteln, Hyperkinese), Bruxismus, Agitation, Schlafstörungen und hohe Schmerztoleranz	Multidisziplinäre Versorgung, regelmäßige psychiatrische Diagnostik
**Angelman-Syndrom**<br>*(Dagli et al. 2025)*	*Pathogene SNVs in UBE3A* (18 %),<br>Matern. Delet. *15q11.2–q13* (62 %)<br>paternale uniparent. Disomie *Chr. 15* (16 %)<br>Imprinting-Defekt *15q12–q13* (4 %)	Ataxie, breitbasiger Gang, Intentionstremor, epileptische Anfälle beginnen meist vor dem 3. Lj., atypische Absencen, myoklonische und atonische Anfälle, sowie generalisierte tonisch-klonische Anfälle	ID/schwere Entwicklungsverzögerung (ab 6 -12 Monaten), scheinbar “fröhliches Wesen“, häufiges Lachen, hypermotor. Verhaltensweisen, Faszination für Wasser/spiegelnde Oberflächen, schwere Schlafstörungen	Multidisziplinäre Versorgung, nonverbale Kommunikationsunterstützung (z.B.Bildkarten), Verhaltenstherapie

SNV=single nucleotide variant (Einzelnukleotidvariante); GATOR= GAP Activity Toward Rags (Regulator des mTOR-Signalwegs); DEE = Entwicklungsbedingte und epileptische Enzephaloapthie; ID= Intelligenzminderung; ASS = Autismus-Spektrum-Störung; ADHS = Aufmerksamkeitsdefizit-/Hyperaktivitätsstörung; FCD = Fokale kortikale Dysplasie; ASM = anfallssuppressive Medikation
